# The iPad as a Research Tool for the Understanding of English Plurals by English, Chinese, and Other L1 Speaking 3- and 4-Year-Olds

**DOI:** 10.3389/fpsyg.2016.01773

**Published:** 2016-11-22

**Authors:** Nan Xu Rattanasone, Benjamin Davies, Tamara Schembri, Fabia Andronos, Katherine Demuth

**Affiliations:** ^1^Department of Linguistics, Macquarie University, SydneyNSW, Australia; ^2^Center for Language Sciences, Macquarie University, SydneyNSW, Australia; ^3^ARC Center of Excellence in Cognition and its Disorders, Macquarie University, SydneyNSW, Australia; ^4^Toybox Labs, Sydney, NSWAustralia

**Keywords:** iPads, preschools, early child second language learning, plural inflectional morphology, Chinese-speaking children

## Abstract

Learning about what young children with limited spoken language know about the grammar of their language is extremely challenging. Researchers have traditionally used looking behavior as a measure of language processing and to infer what overt choices children might make. However, these methods are expensive to setup, require specialized training, are time intensive for data analysis and can have considerable dropout rates. For these reasons, we have developed a forced choice task delivered on an iPad based on our eye-tracking studies with English monolinguals (Davies et al., 2016, under review). Using the iPad we investigated 3- and 4-year-olds’ understanding of the English plural in preschool centers. The primary aim of the study was to provide evidence for the usefulness of the iPad as a language research tool. We evaluated the usefulness of the iPad with second language (L2) learning children who have limited L2 language skills. Studies with school aged Chinese-speaking children show below native performance on English inflectional morphology despite 5–6 years of immersion (Jia, 2003; Jia and Fuse, 2007; Paradis et al., 2016). However, it is unclear whether this is specific only to children who speak Chinese as their first language (L1) or if younger preschoolers will also show similar challenges. We tested three groups of preschoolers with different L1s (English, Chinese, and other languages). L1 Chinese children’s performance was below both English monolinguals and children speaking Other L1 languages, providing evidence that English inflections are specifically challenging for Chinese-speaking children. The results provide further evidence to support previous eye-tracking findings with monolinguals and studies with older bilinguals. The study provides evidence for the usefulness of iPads as research tool for studying language acquisition. Implications for future application of the iPad as a teaching and intervention tool, and limitations for the method, are discussed.

## Introduction

One of the challenges in language acquisition research with toddlers and preschool children is creating age-appropriate and engaging experiments. Young children are limited in both their cognitive and linguistic capacity to follow instructions and maintain attention. Therefore, researchers working with very young children have traditionally relied on analyzing children’s looking behaviors as a proxy for assessing the acquisition of grammar. One such method used to examine early linguistic representations is the intermodal preferential looking (IPL) paradigm (see Golinkoff et al., 1987). In a typical IPL task, children are presented with two pictures side-by-side on a screen. After some time to familiarize themselves with the pictures, children are then played an auditory instruction which matches one of the two pictures. Looking behavior is then analyzed before and after hearing the auditory instruction, which reveals children’s comprehension of the linguistic structure being tested. For example, when testing children’s understanding of nominal plurals, one might show a picture with a single novel object (singular picture) and another picture with five identical new novel objects (plural picture). Upon first viewing the pictures, children’s looking behavior should be random. However, if children understand plural morphology, they should increase looks to the plural picture after hearing auditory instructions such as ‘*look at the teps.*’ Originally, test sessions were video recorded and children’s looking behaviors were manually coded frame by frame in a labor-intensive process. Today, many studies are being conducted using an eye-tracker, where the recording and processing of data can be largely automated. However, we still lack knowledge about what overt choices young children might make on such a task, and how this might relate to looking behavior. Children often show behavioral responses that do not match their looking behavior when they are developing early sensitivities to linguistic structures (Sekerina et al., 2004). Even less is known about the performance of 3- and 4-year-olds on these measures, when the ability to understand and follow instructions is only beginning to emerge (see Trueswell et al., 1999; Sekerina et al., 2004, for studies with older children).

Eye-tracking studies often have considerable dropout rates of 10–50%, depending on the task and ages of the children been tested (Kouider et al., 2006; Mulak et al., 2013; Davies et al., 2016). This can lead to skewed and unrepresentative data. Furthermore, laboratory based studies often have low participation rates, since coming into the lab is not feasible for many busy working parents. There has therefore been a need to find an alternative testing paradigm whereby large numbers of children can be tested quickly with low dropout rates. To ensure high rates of participation, it would be ideal to develop a reliable method for testing children outside of the laboratory at preschools and schools. In recent years, there has been widespread acceptance of touch pad technology, including with young children, who seem to have a good understanding for the concept of making a choice by touching a picture. The touch pad is also extremely portable and easy to use. Furthermore, children appear to be interested in engaging with the touch pad. This is especially important for young children with very limited attention spans; keeping them engaged is an important part of any experimental design. Given these obvious advantages in using the touch pad as a research tool, we developed a series of studies that aimed to replicate IPL and eye-tracking studies on the Apple iPad to test children in preschool settings. In the series of studies reported here, we tested the acquisition of nominal plural morphology by English-speaking monolinguals and Chinese-speaking children learning English, as well as children who speak a variety of different L1s other than English and Chinese.

The acquisition of nominal plural morphology has attracted attention in research with young children as one of the earliest acquired aspects of inflectional morphology in English (followed by present and past tense; Berko, 1958; Brown, 1973; de Villiers and de Villiers, 1973). Adult speakers of English know that the plural *cats* can be decomposed into the root stem *cat* and the plural morpheme -*s*. They are aware of morphological variants of the plural, i.e., the plural morpheme in *cats* is /s/, a voiceless fricative, in *dogs* it is /z/, a voiced fricative, and in *horses* it is /əz/, a full syllable. While the use of plural morphemes in obligatory contexts has been reported in the speech of 2-year-olds (Brown, 1973; de Villiers and de Villiers, 1973), testing their productive knowledge of plural morphology has been challenging. Many preschool aged children are unable to perform the wug task, e.g., presenting the singular stem *wug* and asking children to provide the plural form *wugs* (Brown and Berko, 1960; but see Zapf and Smith, 2007). For this reason, many researchers have used the IPL paradigm to test children’s acquisition of plural morphology. Using this paradigm, one study found that both 2- and 3-year-olds show an understanding of plurals, as indicated by increased looks to the corresponding singular/plural picture after hearing the auditory instructions, e.g., “look there are some *blickets*” (Kouider et al., 2006). What is unclear is whether these children are using other plural cues, e.g., the copula *is/are* or the determiner *some* rather than nominal plural inflectional morphology (-*s*) to perform this task. To test this, the same aged children were given only the nominal inflectional morphemes, “look at the *blickets*,” and only 3- but not 2-year-olds increased looks to the plural picture (Kouider et al., 2006). The results suggest that a full understanding of nominal plural inflectional morphology is acquired late, but that there might be differences in children’s sensitivity to the different plural allomorphs, e.g., /s/, /z/, and /əz/. A recent study addressed this question by testing 2-year-olds with the plural allomorphs /s/ and /z/ (Davies et al., 2016). The results showed that 24-month-olds *do* demonstrate an understanding of plural inflectional morphology, but only for the voiceless fricative plural allomorph /s/ and not the voiced fricative /z/, e.g., *teps* but not *degs*. A follow up study examined the acquisition of the syllabic plural /əz/ (e.g., *tizzes*) and found that 36- but not 30-month-olds show sensitivity to this allomorph (Davies et al., under review). Together these studies suggest that the acquisition of English nominal plurals is a gradual process, with some allomorphs (/s/) acquired earlier than others (/z, əz/). Understanding that *tep* refers to a single object also emerges at around 3-years, suggesting that the grammatical understanding of singular vs. plural morphology develops during the 2–3-year-old period.

These results from monolingual children provide an important baseline for assessing the grammatical development of bilingual and early child L2 (ECL2) learners. Several recent studies of ECL2 learners report continued challenges in using inflectional morphology after many years of exposure to English. For example, Paradis et al. (2016) found that Chinese-speaking children who began learning English at the age of 4 years continue to show difficulties with inflectional morphology after 6 years of English exposure. Some of the structures tested include tense inflections, e.g., past tense ‘*she cooked*,’ and third-person singular -*s*, e.g., “*she cooks now.*” These results are consistent with studies on older Chinese Mandarin-speaking children who began learning English at school (Jia, 2003; Jia and Fuse, 2007). Jia (2003) concluded that some children were unable to attain monolingual-like usage of plurals or tense marking even after 5 years of exposure. In contrast, studies with children from other L1s, including Turkish, Spanish and Punjabi, show good performance on L2 English inflectional grammar during initial acquisition and over time (McDonald, 2000; Marinis and Chondrogianni, 2010; Paradis, 2011; Blom et al., 2012). However, these languages are rich in inflectional morphology, unlike Chinese. For example, the plural in Chinese is marked with a numeral, a modifier, and a noun [e.g., *one modifier cat* vs. *many (optional modifier) cat*]. In English, plurals are inflected with one of the plural allomorphs -*s* or -*es* (e.g., *cats, horses*). Unlike Chinese, English-speaking children must learn that a plural word (e.g., *cats*) is composed of a stem (*cat*) and a plural morpheme (-*s*). This is not required in Chinese and therefore ECL2 learners might find English inflectional grammar challenging. However, so far there have only been studies comparing L2 children with monolingual controls; no study has directly compared the performance of Chinese and other L1 speaking ECL2 learners on inflectional morphology. This is required to understand the effect of L1 Chinese vs. other L1 languages on L2 English acquisition. In addition, studies on L2 acquisition typically use standardized tests, which provide global measures but are not sensitive to fine-grained information like the gradual acquisition of plural allomorphs.

In this study, we addressed these questions using a cohort of monolingual and ECL2 learners speaking L1 Chinese and other languages. In collaboration with Toybox Labs, a series of studies were designed and delivered on the Apple iPad which were based on laboratory based eye-tracking studies (Kouider et al., 2006; Davies et al., 2016, under review). The main aim of the study was to evaluate the usefulness of the iPad as a language research tool, especially with ECL2 learners who have limited L2 English abilities. In order to be a useful research tool, it must have reasonable inclusion rates compared to laboratory-based studies and sensitive for measuring children’s understanding of linguistic structures, e.g., plural morphology. These evaluations are essential and timely because the iPad is portable and easy to use, and could potentially allow large numbers of children to be tested quickly at preschool centers. The method was applied here for assessing L1 Chinese and other L1 speaking children’s performances on L2 English plural morphology. Based on previous iPad studies we expect that the English monolinguals should perform well above chance on all tasks. Given that 2-year-olds are already showing sensitivity to some plural morphemes, English monolingual 3- and 4-year-olds in this study might show close to ceiling performance. However, both groups of ECL2 learners might show lower and more variable performance compared to English monolinguals. If L1 Chinese constrains the learning of inflectional morphology, then Chinese-speaking children should perform worse than English monolinguals and other L1 speaking children. However, if learning English inflectional morphology is challenging for all ECL2 learners regardless of their L1, then both groups of L1 children should perform worse than English monolinguals. In addition, L1 Chinese children might have better performance on the singular items compared to plural inflected items. This is because singular nouns are not marked with inflections and should be readily acquired by Chinese-speaking children.

## Materials and Methods

### Participants

This study was carried out in accordance with the recommendations of the ‘Macquarie University Human Research Ethics Committee’ with written informed consent from all parents of the child participants. Language history questionnaires containing questions about children’s language exposure, family socio-economic status, parental education and whether they had any hearing or developmental delays, were also collected.

The study accepted all 3- and 4-year-olds with signed consent forms as participants for the study. They were drawn from eight preschool centers around the North Sydney area. A total of 69 children (36 girls, 24 boys) participated in the study. The data from nine of these children were excluded from analyses for attempting less than 70% of the trials (six children), not reporting language background (two children) and a history of hearing loss (one child). Data from the remaining 60 typically developing children were analyzed here.

The children were assigned into three groups based on home language. Twenty-two children spoke only English at home and had a native English-speaking mother. Of these 22 children, 10 reported having exposure to another language for between 0.5 and 5 h per week. Nineteen children spoke Chinese at home and 18 had a native Chinese-speaking mother. Of these 19 mothers, 12 were born in China, 3 in Hong Kong, 2 in Taiwan and 1 in Australia and is a heritage speaker of Chinese. Another 19 children spoke a language other than English or Chinese at home^1^. Of these 19 children, 4 were trilingual. All L2 English children reporting speaking a home language other than English and were exposed to English at the preschool. The length of preschool attendance is therefore used here as the measure for length of exposure to English.

The mean age of the children was 48 months (47.5 months for English, 46 months for Chinese, and 49 months for other languages). On average children had been attending preschool for 23 months (22 months for English, 20 months for Chinese, and 28 months for other languages). As a group, these children attended preschool between 12 and 45 h per week.

The education level for mothers ranged from High School to Postgraduate degrees with the majority having either an undergraduate (28 mothers) or postgraduate degree (26 mothers). The education level for fathers also ranged from High School to Postgraduate degrees with the majority having either an undergraduate (27 fathers) or postgraduate degree (22 fathers). The parents of children from the three groups were similarly represented in their levels of education.

### Design

A within subjects design analogous to 2FC (two alternative forced choice) based on the IPL paradigm was used (see procedure for a full explanation). All children were invited to participate in the entire experiment consisting of three blocks. The three blocks tested children’s understanding of suppletive verbal plural morphology using the copula *is/are*, segmental plural allormorphs /s/ vs. /z/, and the syllabic allormorph /əz/.

To avoid any effects of presentation order on performance, the presentation of the three test blocks were counterbalanced across participants. Pseudo-randomizations for the order of trials was also created within each block. While each block contained the same set of nonce objects/animals across the four versions, each object/animal was depicted only once as a plural target, once as a plural distractor, once as a singular target and once as a singular distractor. Pictures were not yoked so that across the four versions no two object/animals were displayed together in more than one trial. Furthermore, no auditory stimulus item was presented with any object/animal more than once across the four versions, regardless of it being a target or distractor picture.

### Stimuli

#### Auditory Stimuli

Auditory stimuli were recorded in a single session to avoid difference in sound quality. The recordings were conducted in a sound-attenuated room and spoken by a female native Australian-English speaker using a child friendly speech register. Audio was recorded using Cool Edit Pro 2.0 sampled at 48 kHz. Stimuli were recorded as complete utterances with carrier phrases. Stimuli for the copula *is/are* test trials were recorded with the carrier phrases “where are [the X]?” and “where is [the X]?” Stimuli for all other trials were recorded with the carrier phrase “touch [the X].”

For the test trials a total of 72 nonce target words were recorded, 36 of which were singular and 36 inflected for plural. Nonce words had onset stops that are early acquired by English-speaking monolingual children: /n/, /d/, /t/, /b/, /p/, /g/, and /k/ (Smit et al., 1990). Vowels were short Australian-English vowels: /æ/, /𝜀/, /ɪ/, /ɐ/ and /ɔ/ (Harrington et al., 1997). In addition to these nonce words, 11 real words were also recorded. Real words were *fox, ducks, clocks*, and *box* in the copula block; *bat(s), crab(s), mop(s)*, and *pig(s)* in the segmental /s/ vs. /z/ plural block; and *horse(s), rose(s)*, and *bus(es)* in the syllabic plural block. The training block contained five trials with all singular target words: *dog, bird, cat, nug*, and *mib*. **Tables 1–3** contain the nonce words used in the test trials.

**Table 1:** Copula (is/are) test block nonce words (with and without copula).

	Singular		Plural	
No copula	dax	/dæks/	dacks	/dæks/
	gex	/g𝜀ks/	gecks	/g𝜀ks/
	gox	/gɔks/	gocks	/gɔks/
	bix	/bɪks/	bicks	/bɪks/
	nux	/nɐks/	nucks	/nɐks/
	poox	/pʊks/	poocks	/pʊks/
With copula	dap	/dæp/	daps	/dæps/
	doop	/dʊp/	doops	/dʊps/
	gip	/gɪp/	gips	/gɪps/
	mep	/m𝜀p/	meps	/m𝜀ps/
	tup	/tɐp/	tups	/tɐps/
	nop	/nɔp/	nops	/nɔps/

**Table 2:** Segmental plural test block nonce words (segmental plural allomorphs /s/ and /z/).

	Singular		Plural	
Voiceless plural allomorph /s/	dup	/dɐp/	dups	/dɐps/
	bip	/bɪp/	bips	/bɪps/
	tep	/t𝜀p/	teps	/t𝜀ps/
	mup	/mɐp/	mups	/mɐps/
	noop	/nʊp/	noops	/nʊps/
	gop	/gɔp/	gops	/gɔps/
Voiced plural allomorph /z/	pab	/pæb/	pabs	/pæbz/
	tib	/tɪb/	tibs	/tɪbz/
	geb	/g𝜀b/	gebs	/g𝜀bz/
	mub	/mɐb/	mubs	/mɐbz/
	koob	/kʊb/	koobs	/kʊbz/
	tob	/tɔb/	tobs	/tɔbz/

**Table 3:** Syllabic plural test block nonce words (syllabic plural allomorph /əz/).

	Singular		Plural	
/s/-final stem	koss	/kɔs/	kosses	/kɔsəz/
	nass	/næs/	nasses	/næsz/
	poss	/pɔs/	posses	/pɔsz/
	dass	/dæs/	dasses	/dæsz/
	bess	/b𝜀s/	besses	/b𝜀sz/
	giss	/gɪs/	gisses	/gɪsz/
/z/-final stem	niz	/nɪz/	nizes	/nɪzz/
	kez	/k𝜀z/	kezes	/k𝜀zz/
	moz	/mɔz/	mozes	/mɔzz/
	tiz	/tɪz/	tizes	/tɪzz/
	doz	/dɔz/	dozes	/dɔzz/
	paz	/pæz/	pazes	/pæzz/

**FIGURE 1:**
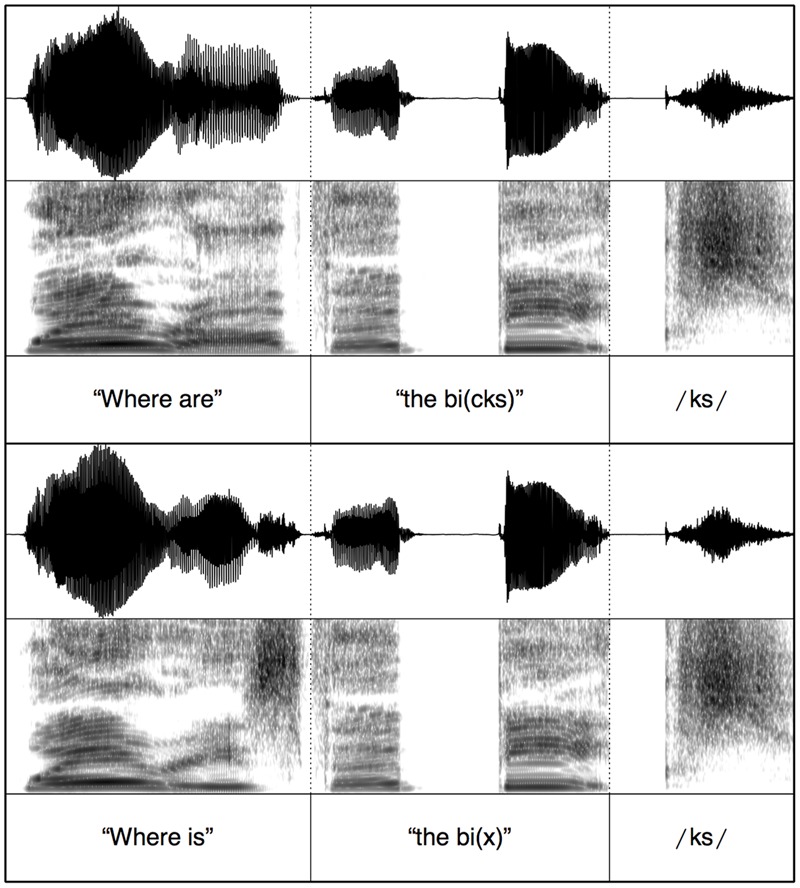
**Example stimulus splicing for auditory stimuli with the copula is/are**.

**FIGURE 2:**
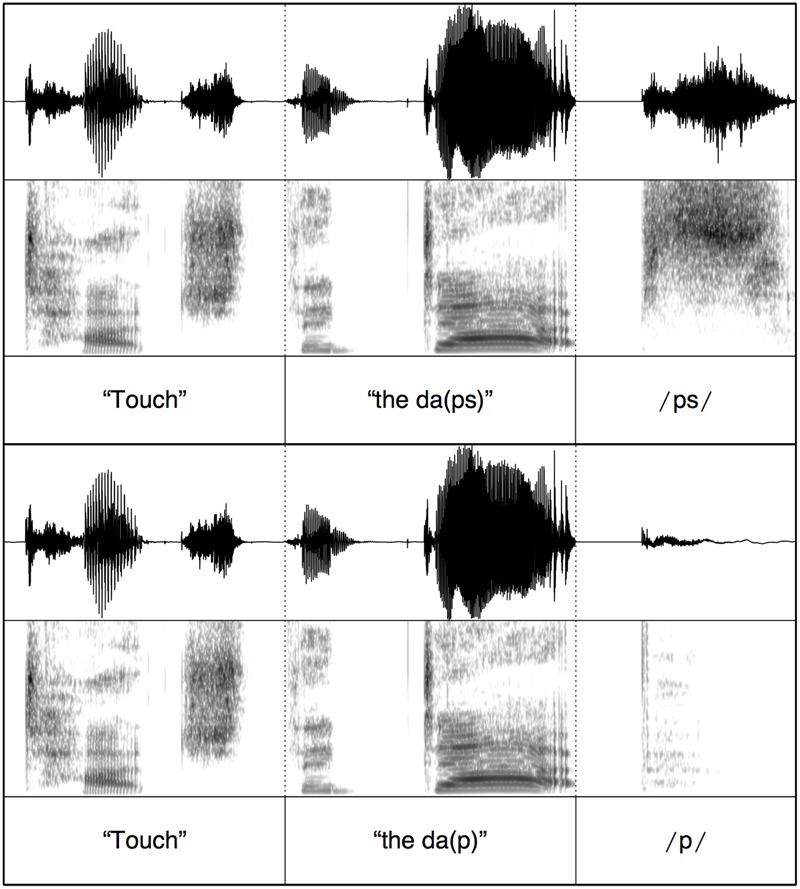
**Example stimulus splicing for auditory stimuli with segmental morpheme /s/**.

**FIGURE 3:**
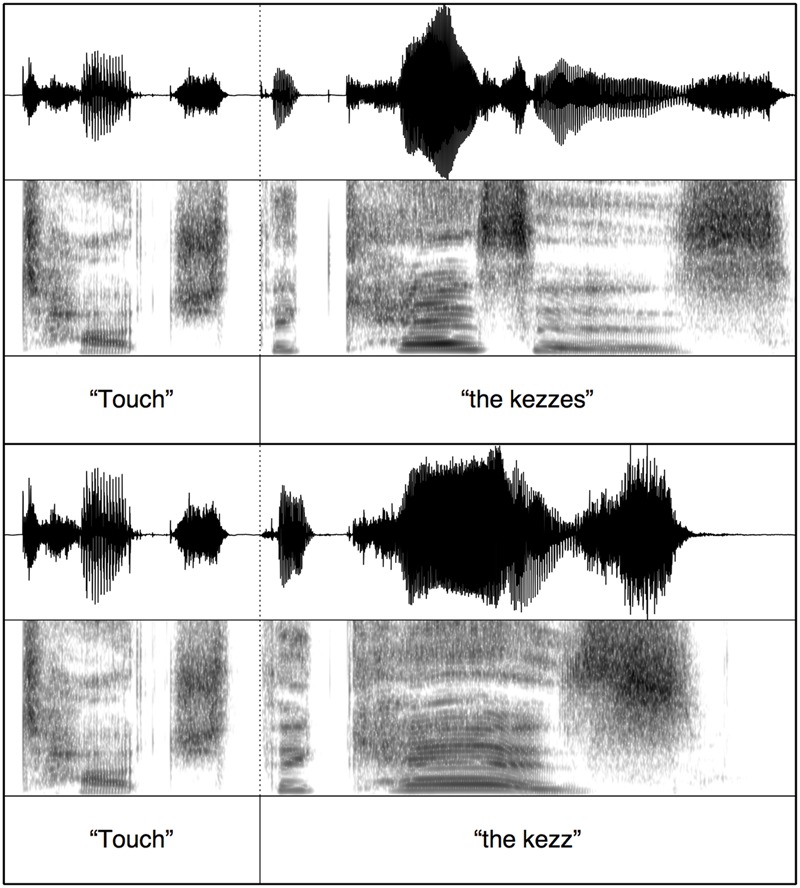
**Example stimulus splicing for auditory stimuli with syllabic morpheme /əz/**.

To ensure minimal acoustic differences across the auditory stimuli, splicing was conducted using Praat (Boersma and Weenink, 2016). For each test block, the target words were spliced onto one carrier phrase. For the copula (*is/are*) test block, the spliced stimuli contained the carrier “where is” + determiner and target word stem (ending at stop closure) + burst release (e.g., /p/ in “dap”) or burst release and frication from the plural morpheme (e.g., /ps/ in “daps”); see **Figure 1**. Therefore, across plural and singular trials the only acoustic difference was the presence vs. absence of the plural morpheme. Stimuli for the segmental plural /s/ and /z/ test blocks were created in a similar way, the only difference being the initial carrier phrase word (“touch”); see **Figure 2**. For the syllabic plural /əz/ test block, the entire target word (singular or plural) was spliced onto the carrier (e.g., “touch” + “the kos” vs. “touch” + “the kosses”). This is done because vowel and frication durations were different in the word stem between the monosyllabic singular (e.g., “kos”) and disyllabic plural words (e.g., “kosses”); see **Figure 3**. These durational differences are naturally occurring between singular and plural real words. The splicing therefore ensured that the stimuli sounded natural.

#### Visual Stimuli

**FIGURE 4:**
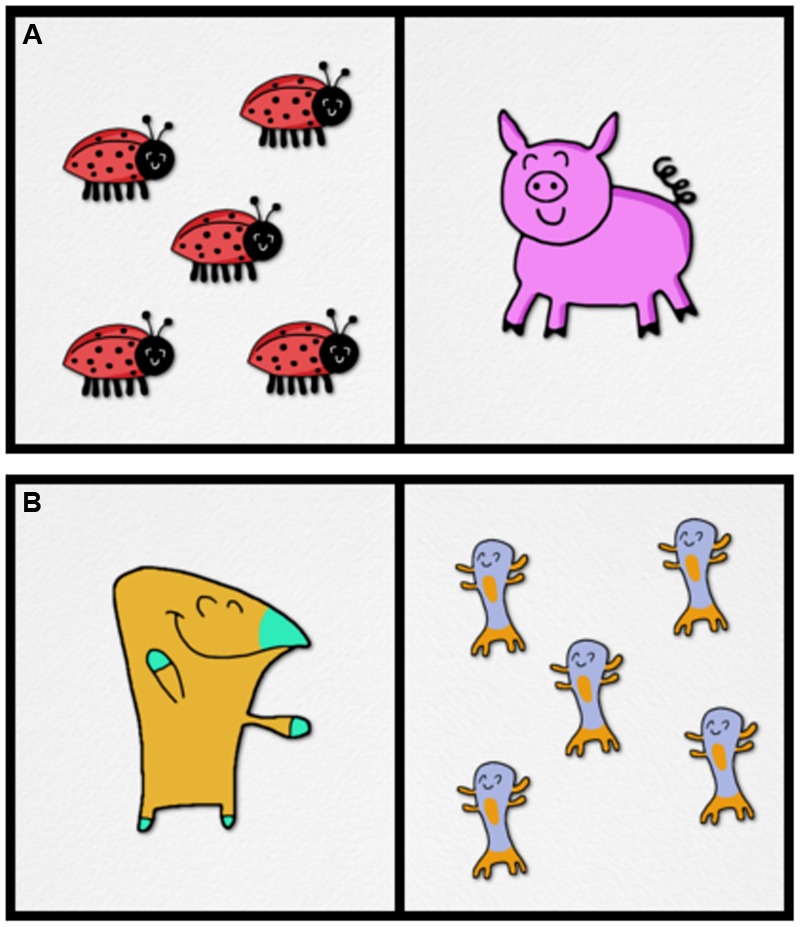
**Examples of (A)** Known animals and **(B)** Novel animals.

Visual stimuli were composed of 24 novel inanimate objects and 48 novel cartoon animals, depicted with happy faces and closed eyes. The novel objects and animals did not resemble anything real or fictional. For known trials, 22 real objects/animals were created. These included *box, shirt, duck, frog, clock, hat, cow, fox, bat, bug, pig, snake, mop, cake, crab, rat, bus, house, rose, tree, horse, and bear*. The known trials were included to maintain children’s interest and were not analyzed. Visual stimuli were constructed as both one object/animal (singular) pictures and five object/animal (plural) pictures. Visual stimuli constructed for the training trials consisted only of singular animals, two of which were novel. **Figure 4** shows examples of a known animal trial (A) and a novel animal trial (B).

### Equipment

The children wore Sennheiser HD 280 pro headphones. The experimental software was built using the Serenity Engine (Budziszewski, 2003) and presented on an Apple iPad Air 2 (240 × 169.5 mm, with a resolution of 2048 × 1536 at 264 dpi).

The Serenity Engine is a multiplatform engine written in C using the OpenGL library. This software makes use of Serenity’s iOS port, with other versions available depending on the situation. Serenity uses the iOS native sound playing capabilities. However, its image displaying capabilities are platform independent. As the current software used a number of large image files, Serenity preloaded the images into memory before each experiment began, ensuring smooth performance throughout. After each trial, results were saved to a text file and then uploaded to an SQL database. As a result, if the experiment was stopped midway, partial results would still be available. If internet access was interrupted, or unavailable during the experiment, results were stored locally on the iPad, and uploaded to the server when internet access was made available. Results were downloaded from a web browser.

The software was designed to allow for a variable number of trials and blocks. These elements can be randomized; alternatively, researchers can pre-specify the order in which items and/or blocks are displayed. Currently, the source code must be manually edited in order to make use of these options. In future, we hope to make these capabilities more accessible to researchers through the use of a scripting language or GUI. This will enable researchers to program experiments which are tailored to their own needs. These will be available on all platforms supported by Serenity. Currently, these are iOS, Windows Phone, PC, Mac, and Linux. The experiments described in this paper will be released on the Apple Store for free, allowing researchers to replicate these experiments.

### Procedure

The children were tested in a quiet area of their preschool, at a child-sized table and chairs. All children wore headphones which helped to focus them to the task, minimized noisy distractions from preschool, and to serve as a blind control for experimenters so they could not hear the stimulus items. The iPad was placed directly in front of the child. To ensure the relevant plural morphemes could be heard, children were first played an /s/ and a /z/ segment extracted from the stimuli. If children indicated they could hear both segments by repeating each sound, the experiment proceeded (if they could not, the volume was adjusted until correct responses were provided).

The initial five trials comprised the Training Block, which tested children’s understanding of the forced-choice paradigm. The training trials presented children with two pictures side-by-side, both depicting a single animal. The first two trials presented the pictures *dog* vs. *cat* and *cow* vs. *bird*. After the pictures had been displayed for 2 s, an auditory prompt told the children to “touch the dog” and “touch the bird.” The third trial presented a *cat* next to a novel animal A, and the child heard “touch the cat.” The fourth and fifth training trials presented children with a *dog* vs. novel animal A, and *bird* vs. novel animal B, and had the auditory stimuli “touch the nug” and “touch the mib.” Upon touching a picture, an audible chirrup would play, and the chosen picture would flash for 1.5 s. This happened regardless of whether the child chose the target or the distractor picture. During the training block, experimenters could give children positive verbal reinforcement if they appeared shy, confused or unsure.

After completing the training trials, understanding of English plural morphology was then tested in the following 47 test trials. For each test trial, two pictures were displayed side-by-side, and after 2 s an auditory stimulus played, encouraging participants to touch one of those pictures. One picture depicted a single object/animal (singular), and the other depicted five different unknown object/animals (plural). The auditory stimulus contained a nonce word that either had a CVC phonological form (e.g., “dup”) to indicate a singular target, or an inflected CVCs/CVCz/CVCəz form (e.g., “teps/degs/kosses”) to indicate a plural target. The use of unknown pictures and nonce words ensured that only understanding of plural morphology was tested and not lexical knowledge.

The 47 test trials were divided into three blocks, each of which tested a different aspect of English plural morphology. Each test block contained trials containing unknown pictures and auditory stimuli, and also known trials, which used familiar pictures and stimuli, in order to help maintain children’s attention toward the task. The copula test block tested children’s understanding of suppletive verbal plural morphology (*is* vs. *are*), and consisted of 16 trials (12 novel, 4 known). The segmental plural test block tested children’s understanding of segmental nominal plural allomorphs /s/ and /z/ (e.g., *tep* vs. *teps*; *deg* vs. *degz*), and consisted of 16 trials (12 novel, 4 known). The syllabic plural test block tested children’s understanding of the syllabic nominal plural allomorph /əz/ (e.g., *koss* vs. *kosses*), and consisted of 15 trials (12 novel, 3 known).

## Results

**FIGURE 5:**
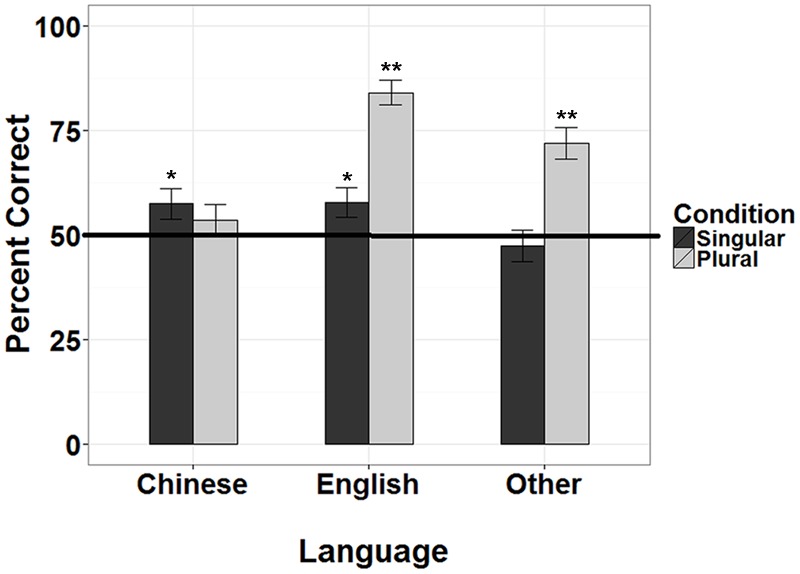
**Percent correct on singular and plural items, with performance compared to chance for three L1 groups (English, Chinese, and other languages).** ^∗^*p* < 0.05, ^∗∗^*p* < 0.01, error bars indicating standard error of the mean and chance at 50%.

To test whether the performance of L1 Chinese children differed from that of the English monolinguals and children speaking other L1 languages, we first conducted *t*-test comparing performance on the singular and plural items against chance for each group (see **Figure 5**). For singulars, both English (*M* = 57.828) and Chinese (*M* = 57.366) children performed significantly above chance [English: *t*(68) = 1.667, *p* < 0.048; Chinese: *t*(36) = 1.688, *p* < 0.047]. For plurals, both English (*M* = 83.907) and children speaking other languages (*M* = 71.930) performed significantly above chance [English: *t*(68) = 1.667, *p* < 0.001; Other: *t*(36) = 1.688, *p* < 0.001]. These results suggest that English monolinguals were performing above chance for both singular and plurals, showing acquisition of plural morphology. Chinese children on the other hand, were above chance only for singular items, while children speaking other L1 languages were above chance only for the plural items.

To examine the effect of L1 and morpheme type on performance, a linear mixed effects regression model (LMER) was conducted in R Core Team (2013) using the *lmerTest( )* function of the *lme4 package* with *Satterthwaite* adjustments to denominator degrees of freedom (Bates et al., 2015). The model included percent correct as the dependent variable with L1 type (English, Chinese, and other languages), Condition (Singular vs. Plural) and Test (Copula, Segmental /s/ and /z/ morphemes, and Syllabic morpheme /əz/) as the fixed factors. Each child was entered as a random variable with random intercept (see **Table 4** for results and R-code).

**Table 4:** Main effects and interaction with estimated values.

Fixed effects	Estimate	Error	df	*t*	*p-value*	Significance
(Intercept)	62.339	6.178	328.716	10.090	0.000	
Main effects					
L1 English	24.024	8.420	330.165	2.853	0.005	^∗∗^
L1 other	10.468	8.721	330.279	1.200	0.231	
Condition (Singular vs. Plural)	-9.613	8.212	303.623	-1.171	0.243	
Test segmental	-12.778	8.111	305.635	-1.575	0.116	
Test syllabic	-13.216	8.111	305.635	-1.629	0.104	
Two-way Interactions					
L1 English × Condition Singular	-31.296	11.136	303.237	-2.810	0.005	^∗∗^
L1 Other × Condition Singular	-21.965	11.531	303.206	-1.905	0.058	ˆ
L1 English × Test Segmental	6.484	11.010	304.695	0.589	0.556	
L1 Other × Test Segmental	6.637	11.459	304.212	0.579	0.563	
L1 English × Test Syllabic	12.140	11.010	304.695	1.103	0.271	
L1 Other × Test Syllabic	16.725	11.459	304.212	1.460	0.145	
Condition Singular × Test Segmental	19.654	11.473	304.425	1.713	0.088	
Condition Singular × Test Syllabic	20.260	11.473	304.425	1.766	0.078	
Three-way interactions					
L1 English × Condition Singular × Test Segmental	7.094	15.609	303.849	0.454	0.650	
L1 Other × Condition Singular × Test Segmental	0.521	16.206	303.603	0.032	0.974	
L1 English × Condition Singular × Test Segmental	-2.517	15.609	303.849	-0.161	0.872	
L1 Other × Condition Singular × Test Segmental	-19.383	16.206	303.603	-1.196	0.233	

*R-code: Lmer (PercentCorrect ∼ L1 ^∗^ Condition ^∗^ Test + (1 | Child)). ˆApproaching significance, ^∗∗^*p* < 0.01.*

A significant main effect for L1 was found, and the ‘L1 English’ term in the model having a positive effect on the intercept suggested that over all English monolinguals performed better than L1 Chinese children, *t*(330.165) = 2.853, *p* = 0.005. There was also a significant L1 by Condition interaction. Further *post hoc* comparisons show that both English children and children speaking other L1 languages performed significantly better on plural (*M* = 83.907 and *M* = 71.930) than singular (*M* = 57.828 and *M* = 47.368) test trials [English: *t*(319.165) = 5.892, *p* < 0.001; L1 other: *t*(318.840) = 5.124, *p* < 0.001]. Not such effects were found for Chinese children. No other significant main effects or interactions were found. This suggests that performance did not differ according to Test type (copula, segmental and syllabic morphemes) for any group of children.

To investigate if age or length at preschool might have any effects on performance, LMEMs were conducted for each language group separately. Test type was removed from this analysis because no main effects or interactions for performance were found in the previous model. Age of the children in months and length of time since starting Preschool in months were added as the fixed variables and subjects remained as a random variable with random intercepts estimated by Condition. **Table 5** presents all main effects and interactions and their estimates as well as the R-code.

**Table 5:** Main effects and interaction with estimated values.

Fixed effects	Estimate	*SE*	df	*t*	*p*	Significance
English						
(Intercept)	44.826	28.206	42.420	1.589	0.119	
Condition	-16.199	40.114	43.580	-0.404	0.688	
Age_mths	0.795	0.585	43.420	1.359	0.181	
Mths_CCC	0.080	0.470	43.590	0.171	0.865	
Condition × Age_mths	-0.088	0.830	44.090	-0.105	0.916	
Condition × Mths_CCC	-0.404	0.666	44.170	-0.606	0.548	
L1 Mandarin						
(Intercept)	6.762	34.308	37.580	0.197	0.845	
Condition (Singular vs. Plural)	88.955	48.446	37.290	1.836	0.074	
Age (months)	1.165	0.716	37.450	1.628	0.112	
Months in Daycare	-0.685	0.728	38.350	-0.941	0.353	
Condition × Age	-2.086	1.011	37.190	-0.640	0.460	
Condition × Months in Daycare	1.122	1.030	38.350	1.090	0.283	
L1 other languages						
(Intercept)	48.742	45.342	38.000	1.075	0.289	
Condition (Singular vs. Plural)	-144.863	64.124	38.000	-2.259	0.030	^∗^
Age (months)	0.045	0.895	38.000	0.050	0.960	
Months in Daycare	1.265	0.628	38.000	2.016	0.051	^∗^
Condition × Age	3.064	1.266	38.000	2.421	0.020	^∗^
Condition × Months in Daycare	-1.809	0.887	38.000	-2.039	0.049	^∗^

*R-code: Lmer (PercentCorrect ∼ Condition ^∗^ (Age + Preschool) + (1 | Child:Condition)). ^∗^*p* < 0.05.*

**FIGURE 6:**
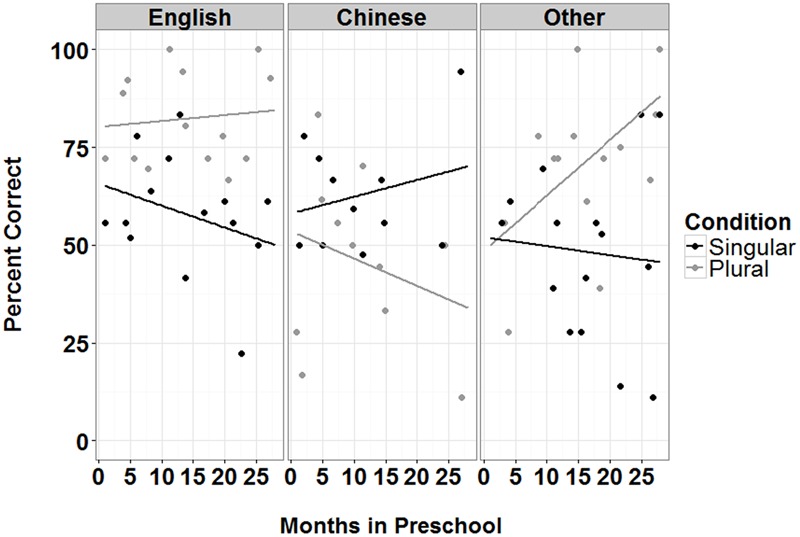
**Percent correct on singular and plural items as a function of months spent in Preschool for three L1 groups (English, Chinese, and other languages)**.

**FIGURE 7:**
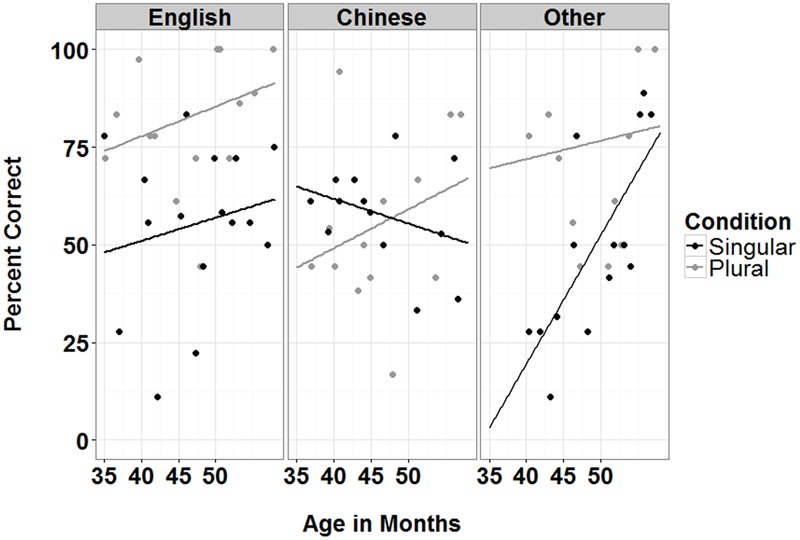
**Percent correct on singular and plural items as a function of Age (months) for three L1 groups (English, Chinese, and other languages)**.

For both English monolinguals and L1 Mandarin children, no significant effects were found. For Other L1 speaking children, there were significant main effects of Condition and length in Preschool, as well as significant two-way interactions between Condition with Age and Condition with length in Preschool (see **Figures 6** and **7**). The results suggest that there were greater improvements on performance with Age for singular than plural items. The reverse was found for length in Preschool, with greater improvements in performance for plural than singular items. For Other L1 speaking children, there is a maturation effect for singulars and a length of exposure effect for plurals.

## Discussion

The main aim of this study was to evaluate the usefulness of using the iPad for language research. We applied this technology to investigate whether L1 Chinese-speaking children show a different acquisition pattern of L2 English plural morphology compared to children speaking other L1 languages. The tests were conducted using a novel item forced choice paradigm delivered on the iPad at preschool centers. Three groups of children were tested differing on L1: English, Chinese or other languages. The results showed that English monolinguals performed better than both groups of L2 learners. On examining singular and plural items separately, it was clear that English monolingual 3- and 4-year-olds demonstrated good understanding for plural morphology and were performing above chance on both singular and plural items, with better performance on plurals than singulars. This pattern of better performance was also found for children speaking other L1s but their performance was above chance only on plural items. This was in contrast to L1 Chinese children who were performing above chance only for singular items. L1 Chinese children’s poor performance specifically in plural inflected forms, which is not shared by children speaking other L1s, reveals a specific problem with acquiring L2 inflections. This provides further support for the findings with older school aged children in Jia (2003), Jia and Fuse (2007), and Paradis et al. (2016). Our results confirm that challenges in acquiring English inflections are not a general L2 learning phenomenon but is specific to Chinese-speaking children. Their pattern of performance on the singular items suggest that Chinese children have developed good linguistic understanding for the singular but may not yet have decoded the linguistic function of plural morphemes.

The results from this study also suggest both age and length of L2 exposure effects for English L2 learning. For children speaking other L1 languages, performance on singular items increased with age, showing a developmental effect. On the other hand, their performance on plural items increased with length of L2 exposure at preschool, showing a L2 learning effect. This result is similar to previous findings from English monolingual patterns of acquisition using IPL/eye-tracking methods, where sensitivity to the /**s/** plural morpheme emerged at 2 years, but sensitivity to the singular form emerged only later, at 3 years (Davies et al., 2016, under review). However, similar effects were not observed in Chinese-speaking children, again suggesting divergent acquisition patterns for Chinese children. While the lack of any developmental or learning effect for L1 Chinese children is concerning, future studies should test older children (5- and 6-year-olds) to avoid any issues with restricted range. More studies with ECL2 learners examining different aspects of language processing, using different perception and production methods, are needed to provide a comprehensive picture of the problem, which has important implications for understanding the processes that contribute to effective L2 language acquisition and processing. Until these studies are conducted, caution must be taken in interpretation these results.

These results have several implications. One implications is for L2 learners of other isolating L1 languages (e.g., Thai), who might show similar challenges in acquiring of inflectional morphology. The expectation is that they might also show poor performance on plural items, similar to that found for the Chinese children in this study. This study was not designed to compare performance in children from different L1 typologies, and therefore does not have the power to address this issue. However, the results suggest that future studies should compare different L1s (isolating vs. inflectional complex) to further our understanding of L2 acquisition. Our findings also have practical implications for teaching L2 English to ECL2 learners, raising the question of whether more targeted training, such as that provided to children with language delay, might ensure faster acquisition of inflectional grammar by Chinese children. To our knowledge, no study has yet attempted any training programs using the iPad to intervene in the process of L2 acquisition. With the high rate of iPad use in young children, more research on the iPad as a useful language-teaching tool should be explored.

In terms of this study’s primary aim, to determine if touch pads us a useful tool for language research, our study provides good evidence for this. We found the iPad to be a very engaging tool for young children. All of the children tested expressed an interest in taking part in the study. In fact, other children who were not tested (could not gain consent from parents) also expressed intense interest in playing with the iPad. We also found reasonable inclusion rates for the children who participated in the study. Of the 69 children who were tested, only six were excluded for attempting less than 70% of the trials – less than 10%. If we took a more relaxed criterion of 50% attempted trials (as is often the case in eye-tracking studies), then only two children would have been excluded. In our experience working with 3- and 4-year-olds, this level of exclusion is very low. A low exclusion rate is useful for several reasons. Most developmental studies with very young children inevitably report data on well-behaved children, with the longest attention span, highest tolerance for boring and difficult tasks and who have eyes that eye-trackers can easily track. Therefore, the data from many typically developing children have not been included in the literature on early development. In this study, where data from almost all of the children are included, we can be more certain that the results are representative of typically developing children. The low exclusion rate also allows data to be collected quickly from a large cohort of children, making it ideal for population level studies. It can therefore be extremely useful in providing much needed data on a range of L2 language acquisition issues and in studying development in general.

In terms of its sensitivity, the method is sensitive enough in discriminating among groups of children with different language abilities. However, given that the English monolinguals were not yet performing at ceiling, there might be developmental effects beyond the ages tested here. This also suggests that a forced choice task might be more difficult compared to eye-tracking. While eye-tracking tasks might reveal early sensitivities to understanding plural morphology, children’s ability to make overt decisions based on their understanding of plural morphology might still be developing at 3 and 4 years. We also did not observe any differences in performance across the different tests involving copula, segmental and syllabic plural morphemes found in eye-tracking studies. This suggests that this type of test may not be as sensitive for addressing fine-grained differences in grammatical knowledge, or might require more trials. Finally, given the low exclusion rate, the iPad task might be suitable for even younger children, i.e., 2 1/2-year-olds.

## Conclusion

The usefulness of the iPad as a research tool was evaluated by testing three groups of children with different L1s (English monolingual, Chinese, and other languages) on their knowledge of plural inflectional morphology. The results suggest that L1 Chinese children’s performance was different from English monolinguals and children speaking other L1 languages. Specifically, L1 Chinese children show difficulties with plural inflected items, suggesting challenges in acquiring inflectional morphology. The results also revealed both developmental and learning effects for children speaking other L1 languages. In using the iPad we found that children were engaged, leading to lower dropout rates, is appropriate for use with ECL2 learner with limited English skills, and the results were sensitivity enough to reveal group differences in performance. This provides evidence for the usefulness of the iPad as a language research tool. Ideally, larger scale longitudinal studies with children of different L1s is required to provide a robust developmental picture of ECL2 acquisition. Perhaps now, with the use of the iPad, researchers can reach more children in preschool centers, providing population level and/or longitudinal developmental data on L1 and L2 language acquisition.

## Author Contributions

All authors contributed to designing the experiment and to the various drafts of this paper. The first, second and fourth authors collected the data. The first author was responsible for the data analysis, interpretation of the data, drafting the paper and updating various versions of the drafts.

## Conflict of Interest Statement

The authors declare that the research was conducted in the absence of any commercial or financial relationships that could be construed as a potential conflict of interest.

## Footnotes

1 Languages reported include: Afrikaans, Armenian, Bengali, Farsi, Filipino, German, Gujurati, Hindi, Italian, Japanese, Korean, Kurdish, Malayalam, Marathi, Polish, Punjabi, Romanian, Serbian, Sinhala, Spanish, Swiss German, Tamil, Thai, Turkish. Of these languages, only Thai is an isolating language like Chinese.
